# Abstracts from Foreign Journals

**Published:** 1902-08

**Authors:** S. J. J. Harger

**Affiliations:** Philadelphia, Pa.


### ABSTRACTS FROM FOREIGN JOURNALS.

### FRENCH.

UNDER THE DIRECTION OF S. J. J. HARGER, V.M.D.,
PHILADELPHIA, PA.

GASTRO-INTESTINAL STRONGYLOSIS IN RUMINANTS. Stiles, after experimenting with various medicinal agents against round worms in the rennet and intestines of sheep, recommends a 1 per cent. watery solution of creosote—60 to 150 grammes, according to the age—to which is added from 2 to 6 grammes of thymol. One administration after twenty-four hours of fasting is usually sufficient. At the end of a week the dose may be renewed.

The author also refers to the method for drenching sheep. The drench is liable to pass into the trachea if the nose is held on a level above that of the eyes. Four methods of drenching were used: The animal standing, sitting on the haunches and held between the legs of an assistant, lying on the side, and lying on the back on an incline. The first two methods were preferable because most of the liquid, unless the animal struggled much, passed directly into the fourth stomach; in the last two most of the drench first entered the rumen.

Moussue and Maratel had satisfactory results with areca nut and extract of male fern, the latter being the most efficacious. Ten grains of the oleoresin of male fern were given, followed by a laxative of sodium sulphate. Eight days afterward no ova of the parasite could be found in the excrement. The flock began to improve at once and, after slaughtering, no strongyli were found in the intestinal tract.—*Rec. de Med. Vet.*

INOCULATION OF CANCER FROM MAN TO THE WHITE RAT. Mayet, in a note to the Academie de Sciences, relates his experiments upon this point: 54 rats were inoculated into the peritoneum with repeated injections of the non-filtered cancerous juice obtained by maceration in water. In some epithelial nodules were found at the necropsy. Five results were positive; 7 doubtful; 42 negative.—*Rec. de Med. Vet.*

NEW MICROBE OF CANCER. Doyen claims to have found in a certain number of cancers a special microbe, designated by him micrococcus neoformans, which he has isolated and cultivated.

Inoculations with the cultures reproduced the neoplasm. Injections of an extract from these cultures produced an inflammatory reaction at the seat of the neoplasm, which suggests to the author the possible preparation of a curative sera.—*Rec. de Med. Vet.*

Parasitic Theories of Cancers. Borrel, at the International Congress of Medicine in Paris, spoke as follows upon the parasitic nature of cancerous tumors:

1. The *microbic theory* is opposed by histological proof. Cancerous and epithelial tumors are essentially characterized by excessive proliferation of the epithelial cell, which multiplies in the metastatic centres and always reproduces the elementary type of the initial tumor. Pathological growths caused by the action of microbes are tubercles or granulomata.

No known bacterium is capable of causing an abnormal proliferation of epithelial tissue.

2. The *coccidian theory* is based upon the existence of parasites belonging to the group of sporozores, reproducing by means of spores, living in preference in the epithelial cells, which they cause to proliferate so as to form veritable tumors. These elements described by authors are pseudoparasites—epithelial cells with a particular evolution or of intracellular inclusions of round, single, or multiple bodies, especially frequent in glandular epithelium and having only a superficial analogy with the coccidia.

The *blastomycetic theory* is based upon the yeast fungus as the causative agent. If such fungi at times exist in these tumors they are rich in the epithelial cells; fresh media inoculated with such products remain sterile, and animal inoculations with this fungus only produce simple neoformations having the structure of granulomata.—*Rec. de Med. Vet.*

Blood Serum of the Horse in Laparotomies. The conclusions of Petit upon the use of the serum heated to 55° C. in abdominal sections are:

1. Ten c.c. of the serum may be introduced into the abdomen at the end of the operation.

2. An intraperitoneal injection may be made twenty-four hours after the operation.

3. These injections are harmless.

4. By stimulation of the phagocytes the serum of the horse heated to 55° C. may prevent infection in laparotomies.—*Rec. de Med. Vet.*

VACCINATION AGAINST CANINE DISTEMPER. Phisalix has prepared a vaccine with which he has vaccinated from May, 1901, to May, 1902, 1250 dogs. Some of these vaccinations were made upon packs of hounds, while others were isolated cases. Some of these were in contact with other dogs affected with distemper: 37 animals succumbed to the disease—a mortality of 2.8 per cent.; 30 were affected with a benign form of the disease.

Of the 37 that died, 26 contracted the disease after the first injection and 11 only after the second. Of the 30 benign cases, 17 recurred after the first and 13 after the second vaccination.

The author is of the conclusion that vaccination will enable the dog to pass over the period of critical susceptibilty to the disease, and that distemper vaccination has a real value.—*Rec. de Med. Vet.*

PROPERTIES AND COMPOSITION OF THE MILK OF THE ASS. The milk of the ass is characterized by a grayish-blue color, peculiar odor, alkaline reaction, a flocculent precipitate in boiling, and, when fresh, unlike cow's milk, does not give the reaction of starch or of Arnold.

*Composition and Chemical Properties.* 1. The albuminoid corpuscles of the milk of the ass are similar to those in woman's milk; the proportion and chemical properties of the casein, albumin, and globulin are also almost identical. Upon these cow's milk is clearly different. The milk of the ass is further characterized by its richness in absorbable albumin, the absence of nucleo-albumin, the special properties of its casein in the presence of acids, pepsin, gastric juice, etc. 2. In asses' milk the quantity of fat and lecithin is relatively little abundant in comparison with that of cow's milk. This fat is besides quite different from that of woman or the cow. 3. The percentage of sugar is a mean between that of woman and the cow. 4. The quantity of salts is about the same as in the other two. 5. The milk of the ass is more aqueous. 6. After artificial digestion it does not contain paraneucleins, which digest with difficulty, while in cow's milk these products after artificial digestion are very abundant. 7. The colostrum, unlike that of cow's milk, which is rich in casein, is relatively poor in albumin.

It results from these observations that the composition and nature of this milk are sensibly different from that of cow's milk, and rather approach that of the milk of woman.—*An. de Med. Vet.*

